# Good multiple sclerosis (MS) care and how to get there in Canada: Perspectives of Canadian healthcare providers working with persons with MS

**DOI:** 10.3389/fneur.2023.1101521

**Published:** 2023-03-02

**Authors:** Julie Petrin, Ruth Ann Marrie, Virginia Devonshire, Draga Jichici, Olinka Hrebicek, Luanne M. Metz, Sarah A. Morrow, Jiwon Oh, Penelope Smyth, Sarah J. Donkers

**Affiliations:** ^1^School of Rehabilitation Science, College of Medicine, University of Saskatchewan, Saskatoon, SK, Canada; ^2^Department of Internal Medicine, Max Rady College of Medicine, Rady Faculty of Health Sciences, University of Manitoba, Winnipeg, MB, Canada; ^3^Department of Community Health Sciences, Max Rady College of Medicine, Rady Faculty of Health Sciences, University of Manitoba, Winnipeg, MB, Canada; ^4^Department of Medicine (Neurology), University of British Columbia, Vancouver, BC, Canada; ^5^Department of Medicine, Faculty of Health Sciences, McMaster University, Hamilton, ON, Canada; ^6^Department of Neurology, Royal Jubilee Hospital, Victoria, BC, Canada; ^7^Department of Clinical Neurosciences, Cumming School of Medicine, University of Calgary, Calgary, AB, Canada; ^8^Department of Clinical Neurological Sciences, Western University, London, ON, Canada; ^9^Division of Neurology, St. Michael's Hospital, University of Toronto, Toronto, ON, Canada; ^10^Department of Medicine (Neurology), University of Alberta, Edmonton, AB, Canada

**Keywords:** multiple sclerosis, team-based care, multidisciplinary, models of care, Canada, qualitative

## Abstract

**Objective:**

The literature that has examined healthcare access and needs of the multiple sclerosis (MS) population is limited. Currently, no research has engaged healthcare providers delivering services to this population to examine their perspectives on the provision of MS care in Canada. We aimed to summarize what good MS care should look like according to Canadian healthcare providers working with people with MS, and to identify the supports and resources required, within their care setting, to enable this standard of care.

**Methods:**

A qualitative descriptive approach was taken to analyze data from participants who responded to additional open-ended survey questions, within a larger “MS Models of Care Survey” targeting Canadian healthcare providers working with persons with MS.

**Results:**

Currently, a gap exists between what healthcare providers working with persons with MS believe MS care should encompass and what they are able to offer. Participants emphasized that their MS clinics are currently understaffed and patient-to-provider ratios are high, leaving very little time to address the array of healthcare concerns their patients present with. The healthcare providers overwhelmingly described that moving toward multidisciplinary team-based MS care that includes appropriate numbers of MS-trained neurologists, nurses, physiotherapists, occupational therapists, and mental health providers working within one location would be their prioritized approach to comprehensively managing MS care. This model of care enables all professionals to effectively coordinate care and use their time efficiently by only focusing on their area of expertise, all while meeting the needs of their patient in one setting, reducing wait-times and improving overall care.

**Conclusion:**

To meet the care needs of Canadians with MS, the healthcare system must consider standardizing and funding multidisciplinary team-based MS clinics, comparable to Stroke units, which continue to show favorable health outcomes after years of implementation.

## 1. Introduction

Multiple sclerosis (MS) is a chronic, inflammatory, immune-mediated disease of the central nervous system that leads to accrued physical, mental, and cognitive disability over time (1). Worldwide, over 2.8 million people are living with MS, and Canada has among the highest prevalence where 1 in every 385 Canadians will develop MS, most commonly in their early adult life (2, 3). MS is a complex chronic condition that can present with a plethora of signs and symptoms, varied clinical disease trajectories and responses to treatment, and multiple comorbidities that lead to worsening of disability (4).

This complexity extends to the management of MS, which involves the treatment or management of acute relapses, MS-related symptoms and common comorbidities, the prescription and monitoring of disease modifying therapies (DMTs) and their effects, as well as the provision of rehabilitation, self-management supports and education. Therefore, best practice guidelines recommend comprehensive care provided by a multi-disciplinary team (5, 6). This approach to care can address the varied needs of persons with MS (PwMS) by facilitating care coordination and continuity within a team-based setting (5, 6). The composition of the MS care team is also important and should include MS neurologists, MS nurses, rehabilitation professionals, mental health providers, specialists, and family physicians (5, 7).

Although there are some care guidelines (5, 6) and evidence that provides important recommendations on the provision of MS care, large variations and gaps in knowledge exist regarding the application of these guidelines internationally, and in Canada. Recent Canadian work has engaged PwMS to assess their access to, and quality of their MS care. Findings suggested a lack of available neurologists, difficulties getting appointments and long wait times for rehabilitation, mental health services, and specialists, which led to a heavy reliance on family physicians for MS care needs (8, 9). These findings shed some light on the state of MS care in Canada. However, no research has engaged healthcare providers working with PwMS to gain their perspectives on the provision of MS care in Canada. Therefore, we aimed to summarize what good MS care should look like according to Canadian healthcare providers working with PwMS, and to identify the supports and resources required, within their care setting, to enable this standard of care.

## 2. Methods

We recently conducted a survey exploring the perspectives of healthcare providers regarding which models of MS care are best suited to meet the needs of persons with MS in Canada. The quantitative data from the survey have been published elsewhere (10). The present paper used a qualitative descriptive approach (11) to analyze the data from participants who responded to additional open-ended survey questions. This paper is reported according to the Standards for Reporting Qualitative Research (12). The University of Manitoba Health Research Ethics Board and Shared Health approved the study.

### 2.1. Setting

This study was conducted in Canada, a geographically vast nation with pockets of high-density population in urban regions and low-density population in Northern and rural areas of the country. Health care is universal and publicly funded for what are deemed as essential services, encompassing hospitalizations and most physician visits. Generally, services provided from non-physicians including physiotherapists and psychologists are not covered unless they are incorporated within a disease-specific hospital-based program that has received funding, such as an MS clinic or as part of in-patient care. Health care is provincially managed and delivered, creating variations across the country. About 60% of Canadians hold some form of private health insurance which can be used to pay for services that are not covered by the universal health system; however there is often a restrictive cap on claimant allowances per provider, differences based on income levels, and coverage is usually linked to employment (13).

### 2.2. Participant recruitment and survey dissemination

We purposefully recruited healthcare providers practicing in Canada who currently deliver care to PwMS, this included health care providers within the 34 MS clinics across Canada (14), as well as general neurologists. The participants were recruited by creating a survey distribution list from multiple sources, including the medical directors of the provincial MS clinics, the Canadian Network of MS Clinics, provincial college of physician listings, and the American Academy of Neurology member directory.

The survey was developed and managed within REDCap (Research Electronic Data Capture), a secure, web-based software that supports data capture for research (15), hosted at the University of Manitoba. Prior to survey distribution, members of the Canadian Network of MS Clinics were advised by email that a survey would be distributed. The survey was then delivered by email to the individuals on the distribution list. The survey was followed by five email reminders. The survey was open and collected data from mid-September 2021 to January 31, 2022. Further details regarding the recruitment and survey administration can be found elsewhere (10).

The survey closed with two open-ended questions. Participants were asked to describe what they thought MS care should look like to best meet the needs of PwMS and what resources would be most helpful in improving MS Care in their respective clinics.

### 2.3. Data analysis

All answers to the open-ended questions were uploaded in Nvivo12 (QSR International Pty Ltd, Melbourne, Australia) a qualitative management software for analysis. The responses were analyzed using content analysis consistent with a qualitative descriptive approach (11). Content analysis is often used in analyzing open-ended survey response, as it remains closest to the original data without adding in interpretations (16). Preliminary analysis of the responses was independently completed by 2 researchers (JP and SJD) with experience in MS qualitative research and with MS care. Both engaged in reading the responses multiple times, after which keywords were highlighted and assigned descriptive codes. These codes were then further examined for similarities and differences, and then categorized. The researchers (JP and SJD) met on multiple occasions to discuss the coding and categorization and adjusted these, as needed. Finally, these categories were further examined and discussed, which led to the identification of three major themes. A third researcher completed an independent analysis and reviewed the finalized categories and themes to provide additional confirmation and triangulation of the coders. The researchers who completed analysis have additional clinical experience with MS care including physiotherapy, neurology, and lived-experience with MS. Notes about codes and decision-making were kept ensuring a robust audit trail (see Supplementary Table 1) and thorough analytical process (17). The datasets generated and analyzed in the current study are not publicly available due to the possibility of identifying information in the qualitative data. Data can be made available from the corresponding author upon reasonable request.

## 3. Findings

### 3.1. Participant demographics

The larger MS Model of Care Survey was distributed across Canada (10). Of 85 total respondents, 57 (67%) health care providers responded to the open-ended questions and were included in this qualitative analysis. The respondents (*n* = 57) represented 20/34 formally labeled Canadian MS Clinics across eight of ten provinces. Respondents were on average 50 years old (SD = 12), where most identified as female (*n* = 37, 65%). Most of the participants were neurologists (*n* = 38, 66%), with 16 (IQR = 5.5–21.5) median years of MS care practice reported. Other healthcare providers who responded to these questions were MS nurses (*n* = 5, 8.8%), nurse practitioners (*n* = 4, 7.0%), occupational therapists (OTs) (*n* = 3, 5.3%), physiotherapists (PTs) (*n* = 2, 3.5%), physician assistant (*n* = 1, 1.8%), social worker (*n* = 1, 1.8%), neuropsychologist (*n* = 1, 1.8%), and patient care coordinator (*n* = 1, 1.8%). The median caseload of respondents was 450 PwMS (IQR = 280–900). See Table 1 for more information regarding the demographic information of the respondents.

**Table 1:** Characteristics of respondents, stratified according to whether practice includes people with multiple sclerosis (*N* = 57).

**Characteristic**	
	**Mean (SD)**
**Age in years**	50.4 (11.7)
	***n*** **(%)**
**Gender**	
Male	20 (35.1)
Female	37 (64.9)
**Discipline**	
Neurologist	37 (64.9)
Physiatrist	2 (68.4)
MS nurse	5 (8.8)
Nurse practitioner	4 (7.0)
Occupational therapist	3 (5.3)
Physiotherapist	2 (3.5)
Physician assistant	1 (1.8)
Social Worker	1 (1.8)
Neuropsychologist	1 (1.8)
Patient care coordinator	1 (1.8)
**Province of practice**	
Ontario	17 (29.8)
Alberta	10 (17.5)
Manitoba	9 (15.8)
British Columbia	8 (14.0)
Saskatchewan	5 (8.8)
Quebec	5 (8.8)
Nova Scotia	2 (3.5)
New Brunswick	1 (1.8)
**Work setting**	
University hospital	38 (66.7)
General hospital	16 (28.1)
Solo private practice	3 (5.3)
Group private practice	1 (1.8)
Other	1 (1.8)
**Work in formally labeled MS clinic**	
Yes	45 (78.9)
**Age of MS patients managed**	
Adults	52 (91.2)
Children (≤ 16 years)	16 (28.1)
	**Median (p25–p75)**
Number of years post-training involved in MS Care	16 (5.5–21.5)
Percentage of clinical work that concerns MS	75 (33-92)
No. MS patients per week, median (p25–p75)	20 (8–30)
No. MS patients in practice, median (p25–p75)^*^	450 (280–900)

* 3 missing (*n* = 54).

### 3.2. Overview of findings

**Figure 1:**
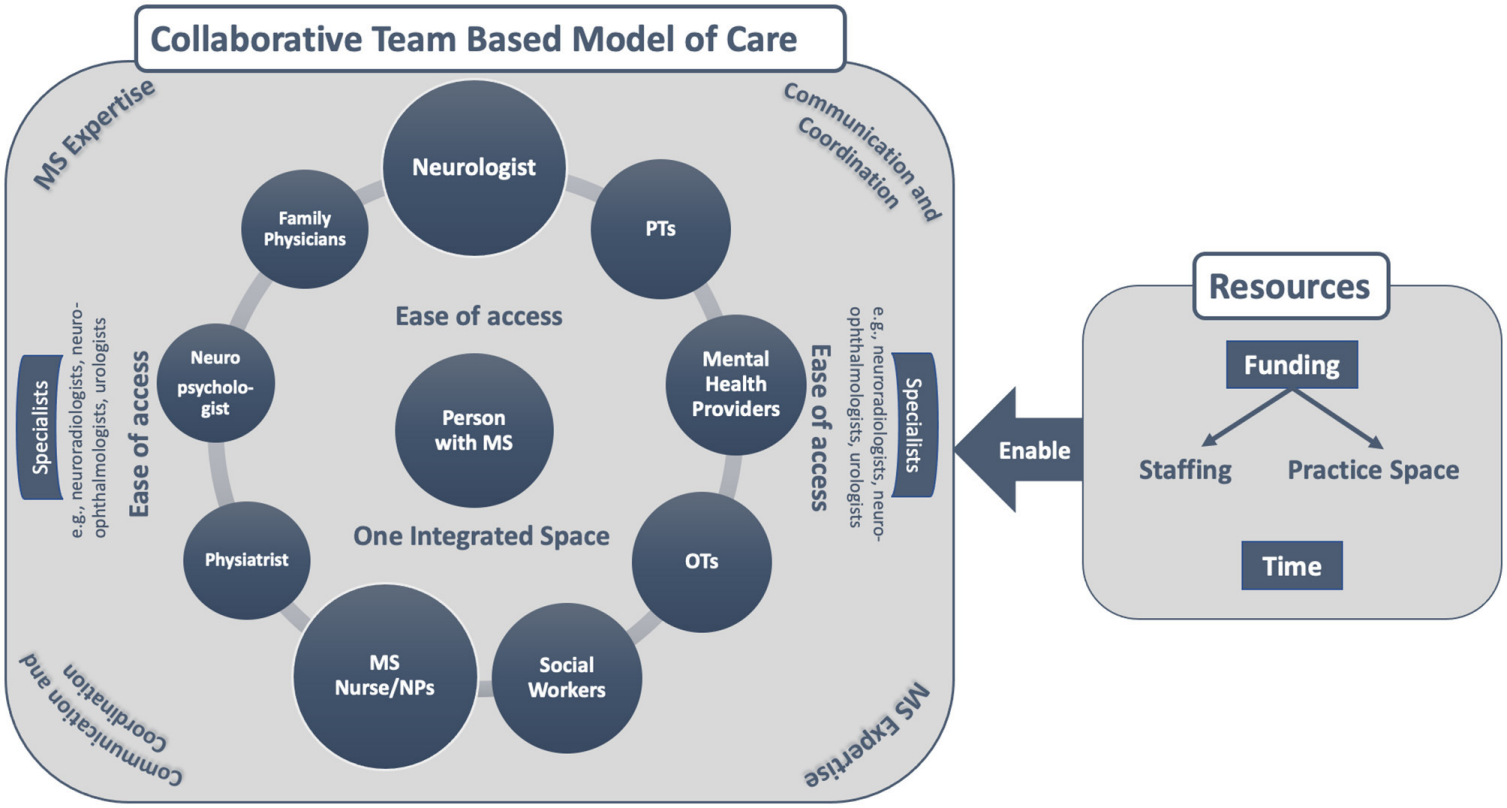
A conceptual depiction of what multiple sclerosis (MS) care should look like according to Canadian Healthcare Providers working with persons with MS as well as the resources required to enable this standard of care. People living with MS, neurologists and nurses play a key role in disease treatment (largest bubbles), but in order to comprehensively manage MS the need for timely access to PTs, OTs, mental health providers and social workers (next largest bubbles) was emphasized. Access to information sharing, smooth referrals, and providers with expertise in MS were highlighted for physiatrists, neuropsychologists, and family physicians. Improved access, communication, and coordination between the team and specialists (neuroradiologists, neuro-ophthalmologists, and urologists) with a level of expertise in MS was also noted.

We identified two main interacting themes: The need for a **team-based approach** to MS care and the corresponding structural, logistical, human, and financial **resources** required to support the development of such a model of care. Participants described a multitude of interconnected components that would lead to better MS care for their patients; however, the only component that was consistently reported across all comments was the need for a “team-based approach” and the resources required to move their clinics toward this approach to care. See Figure 1 for a conceptual depiction of the findings.

#### 3.2.1. Theme 1: Team-based approach

In their responses MS healthcare providers were clear that due to the multifaceted and complex nature of MS therapeutic and symptomatic management it is essential to work as a team to meet their patients' care needs. However, some differences in the terminology being used when referring to this “team-based” approach to care were evident. The most used terms in addition to “team-based” were “multidisciplinary,” followed by “interdisciplinary,” “integrated,” “shared,” and “comprehensive” care. Respondents also had slightly different perspectives on which healthcare providers were essential to this MS Care team. Most agreed that a core team of neurologists and MS nurses/nurse practitioners is necessary for function, however, they further described that to enable a well-functioning clinic, this core team must be complemented with allied care providers, including PTs (most reported) followed by OTs, and mental health providers, including one or a combination of psychologists, psychiatrists, and/or counselors, and social workers. Other providers who were listed as being important team members by multiple respondents were: physiatrists, neuropsychologists (cognitive support in addition to coping and mental health), neuroradiologists, neuro-ophthalmologists, urologists, and family physicians. This is demonstrated by the following answers to the question on what MS care should look like:

“*A multi-disciplinary clinic including PT, OT, social work, neuropsychology, physiatrist, psychiatry, MS nurses and MS neurologists*. (MSC-51, Neurologist, Male, 20 years in MS care).

“*Team approach with efficient access to ancillary services and efficient paper flow with regard to medication access”* (MSC-53, Neurologist, Male, 40 years in MS care).

MS healthcare providers emphasized the importance of not only having a multidisciplinary team but leveraging the benefits of team-based care, including shared care planning by ensuring appropriate communication and coordination of care. Most respondents indicated that working together in one clinic would be the most effective model of care to enable this comprehensive team-based approach. One neurologist working in an MS Clinic stated that: “*ideally multidisciplinary providers are needed within the same clinic, as referrals are cumbersome and delay care”* (MSC-57, Neurologist, Male, 25 years in MS Care). Some described that it was perhaps not necessary to have specialists such as urologists and neuroradiologists on site, however these providers would need to be easily accessible to patients and function as part of the MS care team. Respondents further highlighted the importance of multidisciplinary team meetings to ensure good coordination. This is exemplified by the following quotes:

“*The ideal MS service would be one in which all members of the interdisciplinary team would be available as a resource for the client easily all in one place. That way all members of the team including the client are aware of their health and situation and can work together cohesively.”* (MSC-22, OT, Female, 5 years in MS care).

“*Today the ideal MS service would be a dedicated space to facilitate better comprehensive care and communication” (*MSC-23, PT, Female, 35 years in MS care).

“*A 'one-stop-shop' for our patients and families affected by MS or MS-like conditions in which a single physical site visit can address most if not all active needs.”* (MSC-40, Neurologist, Male, 4 years in MS care).

“*The ideal MS service is with regular multi-disciplinary team meetings”* (MSC-1, Neurologist, Female, 18 years in MS care).

Many respondents highlighted two additional features that were important to a team-based approach to MS care, including: all healthcare providers on the team having expertise in MS and patients having easy access to all team members. Respondents specifically described a need for better access to PT, OT, and mental health. These features are illustrated by the following answers to the questions about what is needed in their current clinic:

“*Multi-disciplinary team preferably with allied health 'dedicated' or well-versed in MS care with rapid access to needed disciplines to maintain/improve quality of life”* (MSC-39, Neurologist, Male, 24 Years of MS care).

“*Greater access to PT/OT/SW/psychiatry/psychology—currently we have very limited access to these professionals and thus they are used sparingly, even though actual need is much greater”* (MSC-54, Neurologist, Male, 15 years of MS care).

“*Significantly more access to psychiatry”* (MSC-13, Neurologist, Female, 21 years of MS care).

Respondents were very clear that to be able to work effectively in a team-based model of care which would meet their MS patients needs the clinics would require resources.

#### 3.2.2. Theme 2: Resources

The most listed resource required to support a team-based approach to MS care across all practices was “*funding.”* Staffing was described by most as a main target for these funds. Most healthcare providers described their practice setting as being short-staffed, where patient-provider ratios were high. Most respondents pointed to the need to staff clinics with more neurologists and MS nurses as well as PTs, OTs, and mental health providers. This is highlighted by the following responses to what is needed in their clinics:

“*Funds to hire inter-disciplinary staff”* (MSC-27, Neurologist, Female, 8 years in MS care).

“*We need more neurologists, nurses, allied health professionals (more FTE) with everyone in a single location for 'one stop shopping.”* (MSC-15, Neurologist, Female, 17 years in MS care).

“*We require a full stock of MS trained neurologist and physiatrists, with on demand, in clinic access to allied health services such as PT/OT/SW/CPAS/SLP/psychology.”* (MSC-56, Neurologist, Male, 5 years in MS care).

Respondents also described a need for support staff that would be responsible for data management and clinical measure assessments. This increase in staffing would be aligned with increased funding to develop and sustain an electronic database that would link patients' electronic medical records to their MS clinical and research data. As demonstrated by the following quotes:

“*We would have assistants to do 9HP and cog/depression screens, 25FTW etc. Need data entry staff to keep up a database.”* (MSC-11, Neurologist, Female, 25 years in MS care).

“*Easy to use database; have a dedicated person that enters and maintains the data professionally”* (MSC-42, Neurologist, Male, 32 years in MS care).

“*Clinical and research database both an EMR and a research quality DB, consistent clinical data”* (MSC-54, Neurologist, Male, 15 years of MS care).

Funding was also deemed necessary to upgrade practice spaces to (1) accommodate the needs of their often mobility impaired patients; and (2) ensure the facilities have the necessary equipment needed by the staff to assess, treat, and manage their patients effectively. As highlighted by the following healthcare providers:

“*Adequate spaces to address patient disability/mobility issues”* (MSC-2, Neurologist, Female, 15 years of MS care).

“*The allied health team would have access to all appropriate durable medical equipment including mobility aids, bathing aids, assistive devices, AFOs etc. to be able to try/demo with patients during assessment to facilitate appropriate prescription.”* (MSC-12, OT, Female, 7 years in MS care).

“*…as well as shared space with appropriate equipment for a variety of interventions.”* (MSC-16, OT, Female, 10 years in MS care).

More funding would enable clinics to be appropriately staffed with the right people, to work effectively as a team in one location, to meet the varied needs of their MS patients. This brings us to the second major resource that was listed, which is *Time*. Time was described as more effectively utilized in team-based approaches, as responsibilities are appropriately shared. With more time, respondents believed that they would be able to more effectively engage in providing comprehensive personalized care, including effective yearly tracking of patient outcomes, patient education and engagement, and personalization of management plans based on needs and goals. As described below:

“*Multidisciplinary care with the time needed for adequate care and education”* (MSC-5, Neurologist, Female, 17 years in MS care).

“*I do spend an hour talking with new patients, and usually a full 30 min face time for follow up, and often they feel that's not enough... I can offer more follow up visits if needed, but I think a supportive team is great for little things that don't need a neurologist would be incredible”* (MSC-7, Neurologist, Female, 6 years in MS care).

In sum, healthcare providers working with PwMS described an MS Care approach that is team-based and driven by patients' needs as opposed to financial or time constraints, which is described perfectly by the following MS patient care coordinator:

“*Baseline screening would be completed on all patients. Time constraints would not be applicable. Interdisciplinary team would be on site. Financial limitations would not lead therapy, treatment, and access to services.”* (MSC-10, MS Patient Care Coordinator, Female, 5 years in MS care).

## 4. Discussion

In this first Canadian study engaging healthcare providers working with PwMS to gain their perspectives on the provision of MS care, the findings were clear. To effectively meet their patients' needs, investments toward supporting the implementation and evaluation of a team-based approach to MS care is required. Our findings point to a gap between what healthcare providers working with PwMS believe good MS care should look like and what is currently happening in practice (e.g., long wait times, lack of multi-disciplinary providers with MS expertise) (10). Respondents described a vision of delivering MS care to work toward, and to further evaluate, in order to improve timely access to comprehensive care. This vision involves MS clinics that are adequately staffed by (or have timely access to) core multidisciplinary providers including neurologists, nurses, PTs, OTs, mental health providers, and social workers, and a model of service delivery that allows providers to work collaboratively to provide timely care within an integrated clinic space. The need for MS care to be team-based has also been endorsed by care guidelines (6, 18) and by PwMS (8, 9, 19). The findings in this study provide insight into what is required to make the necessary changes to reach this standard of care, including, targeted funding toward staffing and clinic space and logistical and organizational shifts to allow for more appropriate time allowances between patients and healthcare providers.

### 4.1. Research in context

Neurologists, the lead healthcare providers in the treatment of MS (20), have been facing challenges in making therapeutic decisions with their patients. Over the past 10 years, the range of DMTs have rapidly expanded, all with varying efficacy and safety profiles (21). Choosing the appropriate DMT, whether to escalate treatment, how to manage and balance the adverse effects of treatment with long-term outcomes are all complex and time-consuming aspects of care. These aspects of care, monitoring disease progression and considering changes in treatments consume a large portion of the time neurologists have with a patient (21, 22). This leaves little time for them to address MS-related symptoms, comorbidities or other concerns including lifestyle changes, which are aspects of care that are considered of high importance by persons with MS (23). These concerns can effectively be addressed through the care of allied health and mental health providers. However, based on the quantitative findings of our study, referrals to these providers lead to long wait times and overall poor access (10), which leaves patients with unmet healthcare needs (8).

Many of the challenges that are faced by healthcare providers and PwMS, such as lack of time during appointments, poor coordination of care, and barriers to accessing care created by limited numbers of healthcare providers and long wait times, may be resolved with the implementation of good multidisciplinary team-based care. Sorensen et al. has been advocating for this model of care, in the form of MS Care units, which he suggests would help mitigate the disease and treatment complexity, shortage of neurologists, poor monitoring of disease activity, low access to timely comprehensive care, discontinuity of care and improve integration of knowledge and shared care that facilitates patient centeredness (20). The current findings should be the last in identifying the need for multidisciplinary care and should move the field toward implementation and evaluation of this model of MS Care.

In moving evidence into practice, it is necessary for all stakeholders to come to a common understanding of multidisciplinary care and how it is situated amongst the other terms referring to these differing models of care. As seen with the participants of our study, the term “multidisciplinary” is commonly used interchangeably with these other terms, and is now used as a catch-all for all models of care that are team-based and not mono-disciplinary (24). There are differences in the extent of collaboration and coordination and focus on patient-centered care between these models, however there are core components that lead to its implementation having improvements on patient outcomes (25). Some of the core components amongst these models of care are: a multidisciplinary team of providers, coordination of care, and patient education and self-management (24). Further to establishing a common understanding of multidisciplinary, the MS community must develop a shared vision and strategy to advocate for funding and policies that enable this movement toward established MS team-based care.

Currently little implementation-based evidence in the MS field has evaluated team-based care. There is evidence that suggests that MS clinics lead to better patient outcomes including fewer emergency room visits (26). However, the quantitative findings from our team's Model of Care Survey have showed that although there is a recognition of the importance a multidisciplinary team-based approach very few clinics fully apply this model in practice. It is rather suggested that these improvements are likely due to the MS expertise of the healthcare providers within the clinics (26), which aligns with the views that our participants had on the need for team members to have appropriate levels of knowledge regarding MS care. One randomized controlled study, in France, compared multidisciplinary care to usual care and did not find any significant differences, other than patient satisfaction, however the authors suggested that it was poor coordination of care and issues in access across providers that may have led to issues in the study (27). Although there is little evidence in the MS field, these models have been implemented successfully for years in the field of stroke care (28) and amyotrophic lateral sclerosis (ALS) (29). In both fields, they have shown that this approach outperforms usual care by improving patient outcomes and satisfaction (28, 29). Treatment of MS is more complex and typically longer lasting, due to its early appearance in life, than both acute stroke and ALS, making the MS population a great candidate for this approach to care (20).

In moving the field toward implementation, it is important to build upon the knowledge that has been generated thus far in this field. It will be necessary for researchers and policy makers to use the successes and failures of previous implementation work and the current identified needs to guide this movement forward. Our findings point to the importance of co-localization of healthcare providers, consistent with findings in stroke and ALS where co-localization improved coordination of care, helped build team rapport and supported the implementation process (28, 30). Further, allied and mental health providers, as well as support staff, were highlighted as key members of the MS care team. This team composition allows providers to focus their time with the patient on their area of expertise, as they know other providers will meet their other needs, which enables each member of the team to engage in more focused patient-centered care (24, 31). General recommendations for the implementation of multidisciplinary care units for chronic illnesses, such as Parkinson's are available to draw upon to help in the implementation stages of this work (31). Further, recommendations on measuring the effectiveness MS Care units (20) are available to help guide these important steps from evidence to practice.

### 4.2. Future directions

There is a continued need to examine the experiences of health care professionals working with PwMS in the provision of care and their perceptions on how to effectively provide care to this population. Researchers need to engage all stakeholders involved in MS patient care, including healthcare providers, persons with MS, health system leaders, and funders, to understand the steps that are required to move multidisciplinary team-based care into practice within MS clinics. In the Canadian context, where healthcare is delivered provincially, leading to differences in care provision, it will be key to implement and evaluate models of care that are federally funded, such as Canadian stroke care units. Future research should focus on evaluating the value of multidisciplinary team-based MS care, by conducting multi-site comparisons of patient outcomes and satisfaction between MS clinics that engage in a multidisciplinary team-based approach to care and those that do not. It would also be important to examine different models of practitioner remuneration to determine the most effective way to promote multidisciplinary team-based care. There are currently comprehensive frameworks that can be used to effectively evaluate team-based care, which can help support these research efforts (32).

### 4.3. Limitations

The major limitation of this work is the inability to gain deeper insights into the answers provided at the time of the survey, as the analysis was completed post-survey. Although we had a large sample of providers across Canada, most were neurologists, which is consistent with most MS Clinics being comprised largely of neurologists, which could affect the findings. These findings are from Canada, although context was provided to ensure researchers could decide the transferability of the findings, it is important to remember that differing challenges are likely across health systems.

## 5. Conclusion

Currently, a gap exists between what healthcare providers working with PwMS believe MS care should encompass and what they are currently offering. Healthcare providers working with PwMS were clear that to meet their patients' needs they need to work toward a model of care that is fully multidisciplinary, which requires resources including funds toward staffing appropriate numbers of Neurologists, MS nurses/nurse practitioners, PTs, mental health providers, OTs, neuropsychologist, social workers and ensuring they have the necessary MS-related knowledge to support their patients. By working in a multidisciplinary team, healthcare providers described being able to spend more time utilizing their unique disciplinary skill sets to manage a patient's multiple complex needs. Further, having appropriately staffed multidisciplinary teams improves access to allied and mental health care. Efforts are needed to move multidisciplinary care into practice to better meet the needs of persons with MS and healthcare providers managing their care.

## Data availability statement

The raw data supporting the conclusions of this article will be made available by the the first author (julie.petrin@queensu.ca) upon reasonable request.

## Ethics statement

The studies involving human participants were reviewed and approved by the University of Manitoba Health Research Ethics Board and Shared Health. The patients/participants provided their written informed consent to participate in this study.

## Author contributions

SJD and JP: conceptualization, methodology, formal analysis, writing—original draft, and writing—review and editing. RM: analysis, conceptualization, methodology, and writing—review and editing. DJ, JO, LM, PS, VD, OH, and SM: conceptualization and writing—review and editing. All authors reviewed the draft, provided feedback, and approved the final manuscript.
