# Supplementary material for: Good multiple sclerosis (MS) care and how to get there in Canada: Perspectives of Canadian healthcare providers working with persons with MS
Source: Front Neurol. 2023 Mar 2;14:1101521. doi: 10.3389/fneur.2023.1101521 (PMC10018146; doi:10.3389/fneur.2023.1101521)
Supplement: Supplementary file 1 [file Table_1.pdf]

Supplementary Table 1. Example Data-Oriented Audit Trail

Individual Quotes	Initial notes	Codes	Categories	Central Themes
A clinic where a client could access multiple providers in one visit- and we could see, and problem solve clients together. (MSC-79)	Collaborative multidisciplinary care	Multidisciplinary approach	Models of Care	Team-based Approach
The ideal MS service would be one in which all members of the interdisciplinary team would be available as a resource for the client easily all in one place. That way all members of the team including the client are aware of their health and situation, and can work together cohesively. (MSC-30)	Collaborative interdisciplinary team in one location	Interdisciplinary care		
Multidisciplinary - integrated to address symptoms including mental health, relapse management, disability/relapse prevention, healthy lifestyle changes, comorbidity - with professionals easily accessible, and sharing the medical record (MSC-23)	Integrated multidisciplinary collaborative care	Integrated multidisciplinary approach		
Adequate spaces to address patient disability/mobility issues. (MSC-62)	Offices that accommodate for appropriate care	Accessible clinical space	Structural Resources	Resources
Add 1.0 FTE RN, 1.0 FTE employee trained to administer the battery of testing, social worker who is part of the team and accessible, an additional 1.0 FTE neurologist specializing in MS. (MSC -53)	More staff required to meet needs	Staffing	Human Resources	
EMR to streamline documentation and communication between team members. (MSC-96)	Effective databases and EMRs	Electronic data management system	Logistical Resources	
Funds for a clinic manager to help organize and manage workflows. (MSC-98)	Funding for support staff	Funding	Financial Resources	
